# CAAFE‐ResNet: A ResNet With Channel Attention‐Augmented Feature Extraction for Prognostic Assessment in Rectal Cancer

**DOI:** 10.1049/syb2.70030

**Published:** 2025-08-27

**Authors:** Qing Lu, Jiaojiao Zhang, Qianwen Xue, Jinping Ma, Sheng Fang, Hui Ma, Yulin Zhang, Longbo Zheng

**Affiliations:** ^1^ College of Mathematics and Systems Science Shandong University of Science and Technology Qingdao Shandong China; ^2^ Qingdao Maternal & Child Health and Family Planning Service Center Qingdao Shandong China; ^3^ Department of General Surgery Penglai People's Hospital of Yantai Yantai Shandong China; ^4^ Department of Pediatrics Affiliated Hospital of Qingdao University Qingdao Shandong China; ^5^ Department of Gastroenterology Surgery Affiliated Hospital of Qingdao University Qingdao Shandong China

**Keywords:** biomedical MRI, feature extraction, medical image processing, neural nets

## Abstract

Magnetic resonance imaging (MRI) has a pivotal role in both pretreatment staging and post‐treatment evaluation of rectal cancer. This study presents an innovative deep learning model, CAAFE‐ResNet18*, based on the residual neural network ResNet18*. The model features an ingeniously designed feature extraction and complementation module (i.e., CAAFE), which leverages a multiscale dilated convolution parallel architecture combined with a channel attention mechanism (CAM) to achieve multilevel information fusion, spatial feature enhancement and channel feature optimisation. This enables in‐depth exploration and augmentation of multilevel downsampled features, significantly improving feature representation capability and overall performance. Testing on rectal cancer MRI data demonstrates that the CAAFE‐ResNet18* model significantly outperforms convolutional neural network (CNN) backbone networks and recent state‐of‐the‐art (SOTA) models. This result indicates that the CAAFE model, by complementing and extracting MR images of patients with locally advanced rectal cancer (LARC) features at different scales from ResNet18*, may help to identify patients who will show complete response (CR) at the end of treatment and those who will not respond to therapy (NR) at an early stage of the treatment.

## Graphical abstract

This paper presents CAAFE‐ResNet18*, an innovative deep learning model leveraging MRI data for prognostic decision‐making in rectal cancer. It outperforms existing models, featuring a plug‐and‐play channel attention‐augmented feature extraction block (CAAFEB) that enhances the model's ability to focus on relevant MRI features and improves feature extraction for identifying disease progression. Experimental validation confirms that CAAFE‐ResNet18* can efficiently and accurately use MRI data, offering clinicians reliable prognostic insights for rapid decision‐making.

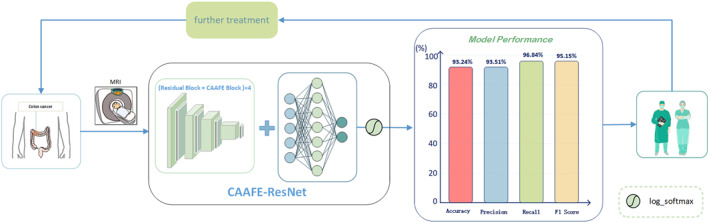

## 1 Introduction

Colorectal cancer is the third most commonly diagnosed malignancy and the second leading cause of cancer death worldwide [1, 2]. Currently, physicians frequently employ magnetic resonance imaging (MRI) [3, 4, 5] techniques in the diagnosis and treatment of rectal cancer. Although MRI is the most accurate imaging modality for primary staging of rectal cancer [6, 7, 8], this is not so for assessing response to therapy. As artificial intelligence has gained widespread attention and applications in medical image recognition [9, 10, 11, 12, 13, 14, 15] and assisted diagnosis [16, 17, 18, 19, 20, 21], we leverage it to develop an innovative deep learning model for the prognostic assessment of rectal cancer. This model will assist physicians in making better therapeutic decisions and improving survival rates for patients with rectal cancer.

Existing deep learning models have achieved significant results in classifying various types of image data and have demonstrated considerable maturity in their applications. For example, Sarwinda et al. [22] employed the ResNet architecture to classify and detect benign and malignant glands in whole slide images (WSI) of colorectal cancer, validating the effectiveness of their approach. This study demonstrates that deep learning techniques exhibit significant reliability and reproducibility in biomedical image analysis. Rengo et al. [23] developed and validated a decision‐support model based on morphological features extracted from MRI using a machine learning approach to distinguish between complete responders and noncomplete responder patients after neoadjuvant chemoradiotherapy, which aligns with the objectives of our study. Popovici et al. [24] utilised a deep convolutional neural network to extract local features from colorectal cancer tissues and combined it with a support vector machine (SVM) classification method to identify molecular subtypes of colorectal cancer. The classifier achieved an overall accuracy of 84% in a multiclass setting and demonstrated significant prognostic value comparable to molecular‐level results, highlighting the potential link between morphological and molecular features. Zhang et al. [25] developed a multimethod integration model, where the improved heterogeneous integration learning model and the generalised kernel recursive maximum correlation entropy algorithm demonstrated superior predictive capabilities compared to support vector machines. Kumar et al. [26] proposed a lightweight convolutional neural network architecture for the automatic classification of histopathological images of various types of colorectal tissues, aiming to enhance the efficiency of early detection of colorectal cancer. Tang et al. [27] proposed a deep learning technique based on MRI for assisted diagnosis. This method not only helps physicians identify metastases more efficiently but also provides a tool for medical imaging students to learn clinical and AI‐related knowledge quickly, thereby enhancing their professional competence and understanding of related technologies.

Although this section of the study reviews several deep learning and machine learning approaches for colorectal cancer, challenges remain. First, the use of the ResNet [22] architecture for pathology slide classification exhibits enhanced feature extraction capabilities; however, it is highly sensitive to the diversity of the dataset and the quality of the labels, which may lead to fluctuations in classification performance. Second, decision support models based on morphological feature extraction from MRI encounter limitations in identifying complex lesions, potentially resulting in misdiagnosis or missed diagnoses that could impact clinical decision‐making. Additionally, deep convolutional networks show significant potential for identifying molecular subtypes; however, their computational complexity and reliance on large‐scale datasets restrict their widespread clinical applicability. Lastly, although lightweight convolutional networks demonstrate advantages in enhancing early detection efficiency, their performance must be further validated to determine whether they can replace traditional methods. Therefore, future research should focus on interdisciplinary method integration, the application of data augmentation techniques and the development of real‐time monitoring systems to address the shortcomings of existing approaches and improve diagnostic accuracy and efficiency for colorectal cancer. However, issues such as excessive model complexity and insufficient clinical applications remain. Additionally, to the best of our knowledge, high‐quality deep learning models that can accurately assist clinicians in making prognostic decisions for patients with rectal cancer are unavailable. Therefore, there is an urgent need to integrate deep learning to help physicians make more efficient and accurate prognoses.

Compared to traditional machine learning methods for prognostic decision‐making in rectal cancer, deep learning approaches effectively circumvent the common feature engineering processes inherent in machine learning [28, 29]. Through the use of multilayer neural network architectures, deep learning automatically extracts and learns complex features from data, significantly reducing reliance on manual feature selection. This capability enables the model to capture intrinsic patterns more effectively when handling large‐scale and high‐dimensional data. Furthermore, deep learning has demonstrated exceptional performance in fields such as image processing, as it excels at capturing nonlinear relationships and extracting valuable information from vast datasets. Notably, residual network (ResNet)‐based architectures excel in computer vision tasks, particularly in global feature extraction. The classic ResNet model, with its unique skip connection structure, not only achieves outstanding performance but also surpasses other state‐of‐the‐art models. This advantage inspires us to leverage the pixel‐level recognition capabilities of the residual neural network in conjunction with MRI data from patients with rectal cancer to facilitate more accurate prognostic decision‐making. Therefore, in this paper, we developed a residual neural network (i.e., CAAFE‐ResNet18*) based on the original ResNet framework to support prognosis decisions for patients with rectal cancer.

The key contributions of this paper are as follows:

1. We designed a deep learning model for prognostic decision‐making in rectal cancer, termed CAAFE‐ResNet18*, aimed at utilising MRI data from patients with rectal cancer to address the urgent need for accurate and timely prognostic assessments in clinical settings. Compared to existing state‐of‐the‐art models, our model demonstrates a significant performance improvement, effectively aiding clinicians in making more precise treatment decisions. By providing reliable prognostic information, CAAFE‐ResNet18* offers robust support for enhancing patient management and optimising treatment strategies.
2. We developed a plug‐and‐play channel attention‐augmented feature extraction block (CAAFEB), which significantly enhances the model's ability to focus on the most relevant features within the MRI data. By incorporating a channel attention mechanism, CAAFEB ensures that the model can dynamically adjust its focus on important features, thereby improving overall feature extraction and representation. This functionality is crucial for distinguishing subtle variations in the imaging data that may indicate disease progression.
3. Experimental results demonstrate the efficacy of the CAAFE‐ResNet18* network, which can efficiently and accurately utilise MRI data from patients with rectal cancer for prognostic decision‐making. This model significantly enhances predictive performance, addressing the urgent need for rapid decision‐making in rectal cancer prognosis. By providing robust prognostic insights, CAAFE‐ResNet18* offers precise decision support for clinicians.

## 2 Related Work

CNNs have achieved remarkable success in the fields of computer vision and medical image analysis in recent years, particularly in automated disease diagnostic systems. CNNs are widely utilised in complex image classification tasks due to their efficiency in extracting deep features. For example, AlexNet [30], by incorporating innovative designs such as local response normalisation and overlapping pooling, significantly enhances the network's ability to learn complex features while improving classification performance. This lays the foundation for the application of deep learning in environments with limited computational resources, particularly in mobile medical devices. VGG [31] significantly enhances model performance while improving feature extraction capabilities by employing a deeper network structure and a small convolutional kernel (3 × 3) design. This architecture promotes the accurate capture of image details and is particularly well‐suited for environments with relatively relaxed computational resource requirements. ResNet [32] introduces residual connectivity, which successfully mitigates the gradient vanishing problem in deep networks, thus driving further deep construction of the model to capture feature information of higher dimensions. This enables significant improvement in accuracy and robustness in image classification tasks. ResNeXt [33] reintroduces the concept of modular design, creating a robust convolutional neural network architecture by employing grouped convolutions and a scalable approach to feature aggregation. This transformation‐based structure effectively enhances the capacity for feature extraction while managing computational complexity, resulting in performance that is competitive with transformer models across various visual tasks. The design optimises feature sharing and increases model flexibility, making it particularly well‐suited for complex computer vision applications. EfficientNet [34] introduces a scaling method that balances depth, width and resolution, enhancing performance across convolutional neural networks (ConvNets). EfficientNet‐B7 [34] achieves competitive accuracy on the ImageNet dataset while being smaller and faster than existing models. It also performs well on datasets such as CIFAR‐100 [35] and Flower [36] with fewer parameters. ConvNeXt [37] reintegrates key concepts from visual transformers into the CNN architecture, enhancing model robustness and flexibility through 7 × 7 deep convolution while achieving performance that is comparable to that of transformers. SCNet [38] leverages a multilayer perceptron (MLP) structure to simultaneously learn both local and overall feature information from medical images. This design effectively integrates features across both spatial and channel dimensions, enabling a more comprehensive capture of key information within medical images, improving classification accuracy and enhancing performance in medical image classification tasks.

Additionally, several attention mechanism models have shown outstanding performance in medical image classification tasks. Through their unique designs and innovative algorithms, these models effectively enhance the analysis capabilities for complex medical images, providing robust support for accurate recognition and classification. For example, Twins [39] focuses on the spatial attention mechanism, significantly enhancing the performance of visual tasks by effectively capturing both local and global features in images. This approach is particularly effective in identifying subtle lesion features in medical image classification. BiFormer [40], on the other hand, employs a two‐layer routing attention mechanism aimed at dynamically selecting relevant features and reducing computational complexity while preserving sensitivity to both global and local features. This design effectively minimises noise interference during the processing of complex medical images, thereby enhancing classification accuracy. In addition, MetaFormer [41] highlights the importance of architectural design by replacing the traditional attention mechanism with a simple spatial pooling operation. This approach has achieved strong results across several computer vision tasks, particularly in medical image analysis, as it maintains high accuracy while reducing parameters and computational overhead. Finally, HiFuse [42] effectively integrates multiscale features through a three‐branch architecture and hierarchical feature fusion, making it particularly well‐suited for addressing complex features and noise in medical images, thereby enhancing the performance of both image classification and segmentation. The innovative design of these models fully showcases the significant potential of the attention mechanism in medical image analysis.

With the advent of ViT [43], the original transformer architecture achieved significant progress in natural language processing, where transformers can capture global contextual information compared to CNNs with localised receptive fields. This advancement has prompted many researchers to explore its application in visual tasks. ViT performs classification by segmenting an image into patches and employing a transformer layer to model the global relationships between these patches. MedViT [44] creates an efficient and robust CNN‐transformer hybrid model by combining the local features of convolutional neural networks with the global connectivity of visual transformers. The model employs efficient convolutional operations to reduce the computational complexity of the self‐attention mechanism and enhances resilience against attacks by adjusting the shape information in the high‐level feature space. Despite the excellent classification results achieved by the ViT model, obtaining large‐scale pretrained datasets remains a challenge for certain applications [43].

The Mamba [45] model has recently made significant advances in the field of natural language processing by employing a transformer architecture. Compared with traditional convolutional neural networks (CNNs), Mamba not only captures global context but also overcomes the computational complexity of visual transformers (ViTs) in capturing long‐range dependencies. By modelling the state space, Mamba efficiently integrates local features and global information, particularly in medical image classification tasks. It significantly improves classification accuracy and robustness while maintaining computational efficiency, driving research progress in related fields. MedMamba [46] is the first visual Mamba model designed for general‐purpose medical image classification. It integrates the capability of convolutional layers to extract local features with a state space model (SSM) to capture long‐range dependencies, aiming to efficiently model various image modalities. Additionally, by employing a grouped convolution strategy and channel shuffling operations, MedMamba achieves fewer model parameters and a reduced computational burden. Numerous experiments [45, 46, 47] validate MedMamba's exceptional performance across a wide range of medical image classification tasks, establishing a new benchmark in the field.

Although several studies have achieved significant results in unimodal medical image classification, there remains a lack of effective prognostic prediction frameworks specifically for rectal cancer. Prognostic prediction in oncology constitutes a critical component of precision medicine, where deep learning techniques have demonstrated substantial potential. In survival prediction, numerous studies have attempted to integrate clinical characteristics, gene expression profiles and imaging data using deep learning models to enhance predictive accuracy. Zhang et al. [48] employed functional principal component analysis to extract longitudinal variations of CEA, CA19‐9 and CA125 levels within 12 months postoperation, incorporating these perioperative longitudinal measurements into a random survival forest model for colorectal cancer survival prediction. Their experimental results demonstrated that inclusion of these temporal biomarkers significantly improved model performance. Gao et al. [49] developed a deep learning system that predicts high‐dimensional gene spatial expression patterns from histopathological images to characterise tumour microenvironments for cancer survival prognosis. In the TCGA dataset's breast and colorectal cancer cohorts, their survival model achieved five‐fold cross‐validated C‐indices of 0.747 and 0.725, respectively, outperforming conventional prognostic models. Specialised prognostic models for rectal/colorectal cancer continue to advance. The DoMore‐v1‐CRC scoring system [50] utilises convolutional neural networks to automatically delineate tumour regions and trains classification models at varying magnification levels, enabling automated survival outcome prediction for patients with early‐stage colorectal cancer undergoing resection.

In this paper, we not only comprehensively compare the classification performance of CNN, ViT and Mamba on a rectal cancer MRI dataset, but also advance the field by designing a CAAFE‐ResNet18* model specialised for prognostic decision‐making. The model is based on the ResNet18 architecture and aims to significantly improve the accuracy of prognostic decision‐making by introducing innovative feature enhancement and mining techniques. This advancement provides new perspectives and methodologies for medical image analysis.

## 3 Data and Methods

### 3.1 Data

**FIGURE 1:**
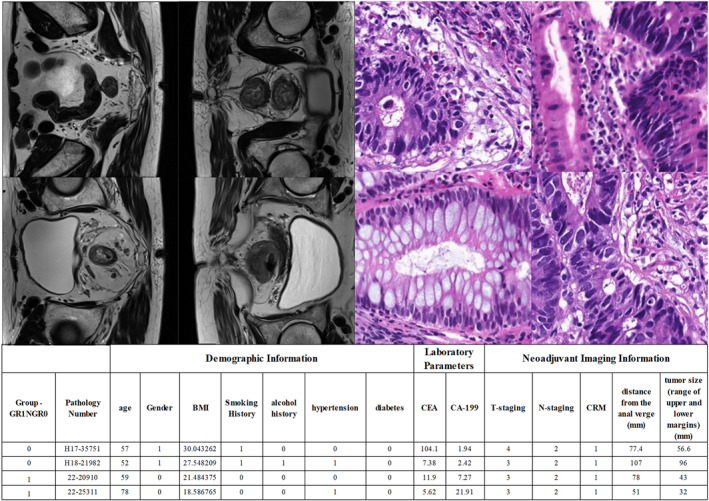
Selected display images of the three types of data are shown, with the four images in the upper left corner representing the raw and sliced 2D MRI images. The image in the upper right corner is a whole slide image (WSI) of the pathology from the tissue biopsy, whereas the image at the bottom illustrates the clinical data.

The data for this study were provided by the Department of Gastrointestinal Surgery at the Affiliated Hospital of Qingdao University, encompassing comprehensive information on 203 patients with rectal cancer. This dataset is divided into three main categories, each derived from the same patient. The first category consists of MRI data, which have been thoroughly annotated by clinicians regarding the tumour site. The second category includes whole slide tissue biopsy images, corresponding one‐to‐one with the MRI data. The third category comprises clinical data, which contain the patient's medical ID, demographic information, laboratory metrics and neoadjuvant imaging information. A partial presentation of the data is illustrated in Figure 1.

**FIGURE 2:**
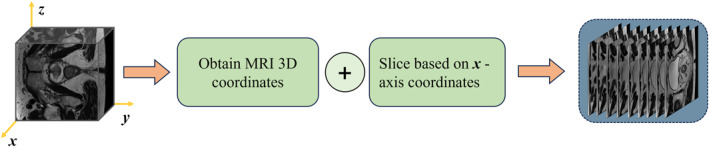
Slicing process of 3D MRI data along the x‐axis.

In the current version of this study, we explicitly did not use whole slide digital section images or any clinical data. This methodological choice arises from the fact that MRI data constitute a fully supervised annotation dataset, whereas the histopathological information carried by whole slide digital section images and the clinical data in textual format exhibit intrinsic disparities in both data structure and annotation paradigms, rendering it infeasible to construct a unified supervised signal integration framework. For the CAAFE‐ResNet18* model, leveraging fully supervised MRI data enables a direct validation of its optimisation efficacy in core capabilities such as discriminative radiological feature extraction and multiscale semantic correlation through a structured annotation system (encompassing dual‐dimensional annotations of pixel‐wise anatomical landmarks and prognostic labels). This approach effectively mitigates evaluation biases in model performance that could arise from annotation heterogeneity across disparate data modalities. We conducted a comprehensive comparison and screening of data from 203 patients with rectal cancer, eliminating invalid data that did not meet the study criteria. After meticulous processing, 185 valid samples were ultimately identified to ensure the reliability and accuracy of the study results. We performed slicing processing on the three‐dimensional (3D) magnetic resonance imaging (MRI) data along the x‐axis. The detailed process is shown in Figure 2. After processing all the available data, a total of 1477 two‐dimensional (2D) cross‐sectional images with a resolution of 256 × 256 pixels were successfully extracted. Among these images, 407 cross‐sectional images are associated with a favourable prognosis, whereas 1070 cross‐sectional images are related to a poor prognosis.

### 3.2 Prognostic Definition and Timeline

This study evaluated tumour response to neoadjuvant chemoradiotherapy (nCRT) as a prognostic indicator, with favourable outcomes defined as either complete response (CR), indicating the disappearance of all tumour lesions, or partial response (PR), characterised by significant tumour shrinkage. Poor outcomes were defined as stable disease (SD), representing no substantial change in tumour size, or disease progression (PD), indicating tumour growth. The primary endpoint was determined based on pathological assessment after curative‐intent surgery.

All baseline MRI scans were acquired prior to the initiation of nCRT, with a consistent interval of 3–4 months maintained between imaging and surgical resection, thereby encompassing the entire clinical course from diagnosis through neoadjuvant therapy to definitive surgery. Prognostic classification was strictly determined by postoperative pathological evaluation and independently verified by two senior clinicians to ensure reliability. This well‐defined temporal framework and outcome classification system provide a robust clinical foundation for subsequent prognostic prediction research.

### 3.3 Model Architecture

**FIGURE 3:**
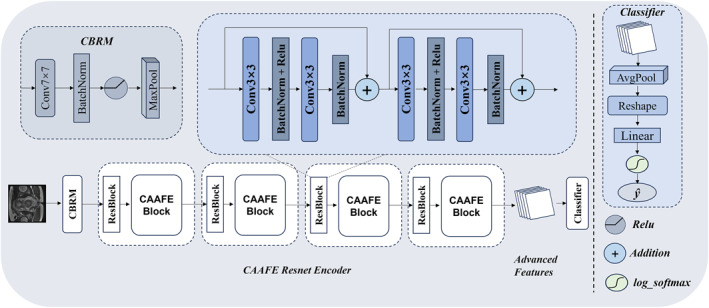
Architecture of CAAFE‐ResNet18*.

The overall framework of the model is shown in Figure 3. The entire design is based on the basic principles of the ResNet18 architecture and is further improved upon. The framework is divided into two main parts: the first part involves feature extraction with the assistance of the channel attention‐augmented feature extraction block, aimed at collecting key features from the input data and performing global feature optimisation and supplementation to maximise the richness of information; the second part is responsible for integrating the extracted high‐level features to generate the final decision results.

#### 3.3.1 Feature Extraction

We trained an efficient feature extractor using a convolutional neural network for the MRI data of patients with rectal cancer. First, we used the CBRM module to perform convolution, normalisation and pooling operations on the input images to capture local features and spatial relationships effectively. In the CBRM module, we employed a 7 × 7 convolutional kernel to retain as much of the original data information as possible. Subsequently, we applied batch normalisation and max pooling to the extracted features to stabilise the feature distribution and accelerate the convergence of training. Through these preprocessing techniques, we effectively prevented overfitting and enhanced the model's generalisation capability and computational efficiency. The computational process of the entire CBRM module is as follows:

(1) y=MaxPool(ReLu(BN(Conv(x)))),

where x is the input data, Conv() denotes the convolution operation with a kernel size of 7 × 7, a stride of 1 and no padding. BN denotes batch normalisation, and ReLU is the activation function.

**FIGURE 4:**
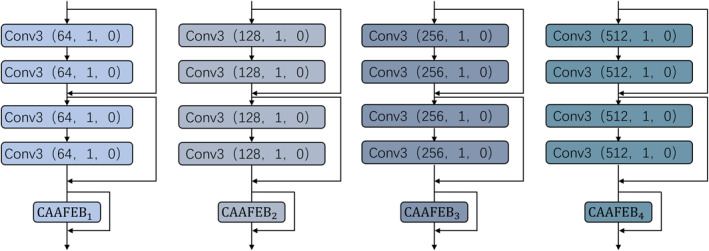
Feature extraction modules.

Subsequently, we designed a CAAFE‐ResNet encoder comprised of four distinct feature extraction and enhancement blocks. This multiscale feature fusion architecture effectively captures multilevel and multigranularity semantic features within images, providing richer and more discriminative feature representations for subsequent decision‐making tasks. Each feature extraction and enhancement block consists of a residual block (ResBlock) and a CAAFE block. Each ResBlock contains two consecutive residual connections, with each module comprising two convolutional layers, batch normalisation, ReLU activation functions and a residual connection structure. As the network deepens, we set 64, 128, 256 and 512 convolutional kernels in the four ResBlocks, respectively. This strategy of progressively increasing the number of convolutional kernels effectively expands the network's receptive field. Furthermore, after feature extraction is completed within each ResBlock, the CAAFE block is employed to integrate global features, maximising feature extraction capability and capturing multiscale visual features ranging from low‐level textures to high‐level semantics. The CAAFE block not only captures local texture information within the image but also emphasises critical interchannel information through a channel attention mechanism, thereby providing global enhancement and supplementation of the features extracted by the ResBlock. The structure of the residual connection can be represented as follows:

(2) $$y={\left(ReLu\left(BN\left(Conv\left(x\right)\right)\right)\right)}^{2}+x,$$

where x is the input feature, Conv() denotes the convolution operation with a kernel size of 3 × 3, a stride of 1 and no padding. BN denotes batch normalisation, and ReLU is the activation function. The architecture of the four feature extraction modules is illustrated in Figure 4.

#### 3.3.2 Channel Attention‐Augmented Feature Extraction Block (CAAFE Block)

**FIGURE 5:**
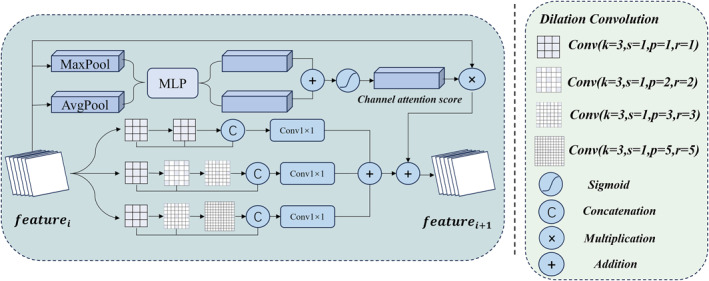
Channel attention‐augmented feature extraction block.

As low‐level features are processed through the ResBlock, the dimensionality of the feature space gradually increases, whereas the scale decreases. This can easily lead to the loss of details in target objects and tissue textures, posing challenges for subsequent decision‐making tasks. To address this issue, we designed the CAAFE block. The CAAFE block utilises diverse receptive fields from multiple dilated convolutions to concentrate spatial information across each channel. In the context of channel information integration, we employ a channel attention mechanism to aggregate interchannel information effectively. This global information collection approach effectively mitigates the loss of details caused by successive downsampling, bridging the gap between low‐level features and high‐level semantic features. Specifically, we utilise convolutional kernels with varying dilation rates to decouple the feature maps extracted from the ResBlock into three parallel feature mapping perspectives. Each mapping employs a distinct feature extraction method, which are subsequently aggregated. This diverse receptive field design not only enhances the representational capacity of the features but also significantly enriches their diversity, thereby enabling a more comprehensive capture of the key information and complex patterns within the data. The upper‐middle section of Figure 5 illustrates the architecture of the CAAFE block.

In the CAAFE block, we designed a parallel structure with three diverse receptive fields to enhance and supplement spatial feature information as much as possible. In the first parallel structure, two 3 × 3 convolutional kernels are employed for continuous feature extraction. Upon completion, the initial features, transitional features and final features are integrated, and 1 × 1 convolutions are utilised to aggregate these features.

In the second parallel structure, we first employ a 3 × 3 convolution to extract features from the initial input, facilitating information interaction with the initial features from the first parallel structure. Upon completion, dilated convolutions with the same kernel size but dilation rates of 2 and 3 are applied to mine the transitional features deeply, enabling the capture of information through multiple receptive fields. Finally, the initial features, all transitional features and final features are integrated, and a 1 × 1 convolution is utilised to aggregate these features. Furthermore, to enhance feature normalisation, the aggregated features undergo a normalisation process.

In the third parallel structure, we also employ a 3 × 3 convolution to extract features from the initial input, thereby maintaining information interaction of the initial features with those from the first and second parallel structures. Upon completion, we expand the receptive field to capture additional critical features. Building upon this, we utilise dilated convolutions with the same kernel size but increase the dilation rates to 3 and 5 for continuous and in‐depth extraction of transitional features, thus enabling a broader capture of spatial information. Finally, after integrating all features, a 1 × 1 convolution is applied to achieve feature aggregation and normalisation.

After the completion of information extraction from spatial features by the three parallel structures, we systematically aggregate the final outputs from each structure. This integration process aims to leverage the diverse feature information extracted through these three distinct pathways, thereby enhancing the model's understanding of spatial features. The final aggregated result not only retains the unique information from each structure but also promotes the complementarity among features, providing a more comprehensive feature representation for subsequent tasks. The three parallel processing pathways can be described as follows:

(3) $$x1=Cat\left[Conv3_{1}\left(f_{i}\right),Conv3_{1}\left(Conv3_{1}\left(f_{i}\right)\right)\right],$$

(4) F1=BN[Conv1(x1)],

(5) $$x2=Cat\left[Conv3_{1}\left(f_{i}\right),Conv3_{2}\left(Conv3_{1}\left(f_{i}\right)\right),Conv3_{3}(Conv3_{2}\left(Conv3_{1}\left(f_{i}\right)\right))\right],$$

(6) F2=BN[Conv1(x2)],

(7) $$x3=Cat\left[Conv3_{1}\left(f_{i}\right),Conv3_{3}\left(Conv3_{1}\left(f_{i}\right)\right),Conv3_{5}(Conv3_{3}\left(Conv3_{1}\left(f_{i}\right)\right))\right],$$

(8) F3=BN[Conv1(x3)],

where $f_{i},i\in \left\{1,2,3,4\right\}$ are the features extracted by the BasicBlock, $Conv3_{r}\left(*\right)$, r∈{1,2,3,5} denotes a convolutional kernel of size 3 × 3, where *r* is the expansion rate. Cat[ ] denotes a concatenation operation. Conv1() denotes a convolutional kernel of size 1 × 1. BN[ ] denotes a batch normalisation operation. Fi,i∈{1,2,3,4} are the results of the four parallel feature mappings from the module.

Building upon the enhancement and extraction of spatial features, we introduced a channel feature supplementation mechanism to further enrich global feature representations and enhance the model's decision‐making capabilities. To achieve this, we employed a channel attention mechanism (CAM) [51] to effectively augment and complement the channel features. This approach not only improves the diversity of feature representations but also optimises the model's performance in handling complex tasks, thereby facilitating the synergistic interaction among features and enhancing overall performance. After completing the information enhancement of both spatial and channel features, we effectively aggregated these two types of features to obtain a richer global feature representation. This aggregation process significantly enhances the expressive power of the features and provides more comprehensive support for subsequent model decisions. By integrating spatial and channel features, we are able to capture the underlying patterns within the data more accurately, thereby markedly improving the model's performance and robustness. This process can be described as follows:

(9) $$F4=CAM\left(f_{i}\right),$$

(10) $$F_{\mathrm{out}}=F1+F2+F3+F4,$$

In the model's end architecture, we constructed a feature‐decision transformation module. By implementing global pooling for dimensionality reduction and linear space mapping on the heterogeneous features output by $CAAFEB_{4}$, combined with logarithmic probability modelling (LogSoftMax), we achieved a diffeomorphic transformation from the feature manifold to the probability manifold. Through a dual mechanism of implicit feature calibration and explicit probability constraints, we established a classification boundary optimisation framework that maintains the differentiability of the decision‐making process while offering interpretability from an information geometry perspective. This process can be described as follows:

(11) $$\hat{y}=\sigma \left(\text{fc}\left(\text{Reshape}\left(\text{AvgPool}\left(z\right)\right)\right)\right),$$

where

z is the result after the output of ${\text{CAAFEB}}_{4}$;

σ(x) is the logSoftMax activation function, defined as follows:

$$\sigma \left(x\right)=\log\left(\frac{e^{x_{i}}}{\sum_{j=1}^{n}e^{x_{j}}}\right)=x_{i}-\log\left(\sum_{j=1}^{n}e^{x_{j}}\right),$$

where $x_{i}$ is the i‐th element of the input vector x, and n is the total number of elements in x;

AvgPool(z) is the average pooling operation applied to z;

Reshape(x) is the reshape operation applied to x;

fc(x) is the fully connected layer applied to x.

## 4 Numerical Experiment and Result Analysis

Because of the unbalanced nature of the data, out of 185 patients, only 48 had a good prognosis, whereas 137 had poor prognostic outcomes. To minimise the issue of insufficient training due to limited samples, we divided 70% of the rectal cancer MRI data into a training set, 10% into a validation set and the remaining 20% into a test set. To prevent overfitting, we applied data augmentation techniques such as random rotations, random horizontal flips and random adjustments of brightness and contrast. Table 1 summarises the general properties of the rectal cancer MRI dataset.

**TABLE 1:** Summary of MRI data for rectal cancer.

Datasets	Total number (sheets)	Train	Validation	Test
MRI data of rectal cancer	1616	1181	139	296

### 4.1 Experimental Details

Our experiments were conducted on an NVIDIA RTX 4060 GPU. The loss function used was cross‐entropy loss, and the optimiser was SGD, with a learning rate set to 0.001, weight decay at 0.00001, momentum at 0.7 and a batch size of 16. A total of 40 epochs were trained. The data were enhanced by randomly flipping and adjusting the image brightness, contrast, saturation and hue, with the input image size adjusted to 256 × 256.

### 4.2 Assessment Indicators

We used the same set of evaluation metrics as most classification methods. These metrics include accuracy, precision, recall and *F*1 score.

Accuracy is defined as the ratio of the number of correctly predicted samples to the total number of samples, reflecting the overall correctness of the model:

(12) $$Accuracy=\frac{TP+TN}{TP+TN+FP+FN},$$

where TP denotes true positive, TN denotes true negative, FP denotes false positive and FN denotes false negative. Accuracy is an intuitive performance metric, but it may be biased in cases of uneven sample distribution.

Precision is defined as the proportion of true positives among the samples predicted as positive by the model, reflecting the model's ability to correctly predict positive cases:

(13) $$Precision=\frac{TP}{TP+FP}.$$

Precision focuses on how accurately the model predicts positive examples.

Recall is defined as the proportion of positive cases correctly identified by the model out of all positive cases, reflecting the model's ability to identify positive cases:

(14) $$Recall=\frac{TP}{TP+FN}.$$

Recall focuses on the model's coverage of positive examples.

The *F*1 score is the harmonic mean of precision and recall, combining the model's accuracy and coverage:

(15) $$F1=\frac{2\times \text{Precision}\times \text{Recall}}{\text{Precision}+\text{Recall}}.$$

The *F*1 score is a balanced assessment metric that is particularly useful when there is a trade‐off between precision and recall.

### 4.3 Experimental Evaluation

#### 4.3.1 Performance Comparison

We compared the performance of the CAAFE‐ResNet18* model with classical classification models such as ResNet18, ResNeXt50, VGG16, AlexNet and EfficientNet on the rectal cancer MRI dataset, as well as with recent state‐of‐the‐art (SOTA) models.

**FIGURE 6:**
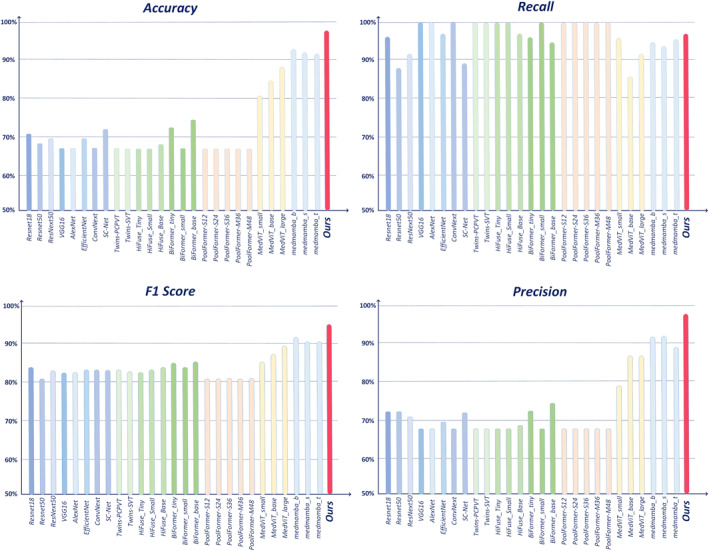
Performance comparison on unimodal rectal cancer MRI dataset.

Table 2 demonstrates the overall performance of CAAFE‐ResNet18* on the rectal cancer MRI dataset, and the results of the comparison experiments are visualised in Figure 6. The results showed that our CAAFE‐ResNet18* method achieved the best performance among all the compared models, reaching 93.24% and 95.15% for accuracy and *F*1 score, respectively. For the rectal cancer MRI dataset, CAAFE‐ResNet18* performed exceptionally well compared to all other models, indicating that our model's performance is highly competitive. Specifically, compared to CNN‐based networks (VGG16, ResNet18, ResNet50, ResNeXt50, AlexNet, EfficientNet, ConvNeXt), it achieved a 21.89% improvement in accuracy over the best‐performing ResNet18 network (from 71.35% to 93.24%) and a 12.7% improvement in the *F*1 metric (from 82.45% to 95.15%). CNN‐based networks primarily focus on local detail information, making it challenging to effectively distinguish between images with similar features. Although CAAFE‐ResNet18* leverages CNN to enhance the extraction of local detail features, it also comprehensively analyses these features across multiple levels and stages, enabling more accurate feature recognition. Experimental results show that compared to convolutional networks that primarily focus on local detail information, our model significantly improved its performance after incorporating the feature supplementation and mining module.

**TABLE 2:** Performance comparison on the rectal cancer MRI dataset, with figures in bold indicating the best result in each metric, including accuracy, sensitivity, precision and *F*1 score.

Category	Model	Accuracy (%)	Precision (%)	Recall (%)	*F*1 score (%)
CNN	ResNet18 [32]	71.35	70.94	98.42	82.45
	ResNet50 [32]	69.19	71.00	92.89	80.48
	ResNeXt50 [33]	69.73	70.20	96.84	81.40
	VGG16 [31]	68.38	68.38	**100**	81.22
	AlexNet [30]	68.38	68.38	**100**	81.22
	EfficientNet [34]	70.00	69.94	98.42	81.77
	ConvNeXt [37]	68.38	68.38	**100**	81.22
MLP	SCNet [38]	71.62	74.18	89.72	81.22
Twins	Twins‐PCPVT [39]	68.38	68.38	**100**	81.22
	Twins‐SVT [39]	68.38	68.38	**100**	81.22
HiFuse	HiFuse_tiny [42]	68.38	68.38	**100**	81.22
	HiFuse_small [42]	68.38	68.38	**100**	81.22
	HiFuse_base [42]	69.46	69.44	98.81	81.57
BiFormer	BiFormer_tiny [40]	71.89	71.35	98.42	82.72
	BiFormer_small [40]	68.38	68.38	**100**	81.22
	BiFormer_base [40]	72.43	71.88	98.02	82.94
MetaFormer	PoolFormer‐S12 [41]	68.38	68.38	**100**	81.22
	PoolFormer‐S24 [41]	68.38	68.38	**100**	81.22
	PoolFormer‐S36 [41]	68.38	68.38	**100**	81.22
	PoolFormer‐M36 [41]	68.38	68.38	**100**	81.22
	PoolFormer‐M48 [41]	68.38	68.38	**100**	81.22
MedMamba	MedMamba_b [46]	88.38	89.47	94.07	91.71
	MedMamba_s [46]	88.11	89.43	93.68	91.51
	MedMamba_t [46]	87.30	87.87	94.47	91.05
MedViT	MedViT_small [44]	78.92	77.96	96.44	86.22
	MedViT_base [44]	82.70	87.06	87.75	87.40
	MedViT_large [44]	85.41	86.99	92.49	89.66
**Ours**	CAAFE‐ResNet18*	**93.24**	**93.51**	96.84	**95.15**

*Note:* The bold values represent the optimal results.

**FIGURE 7:**
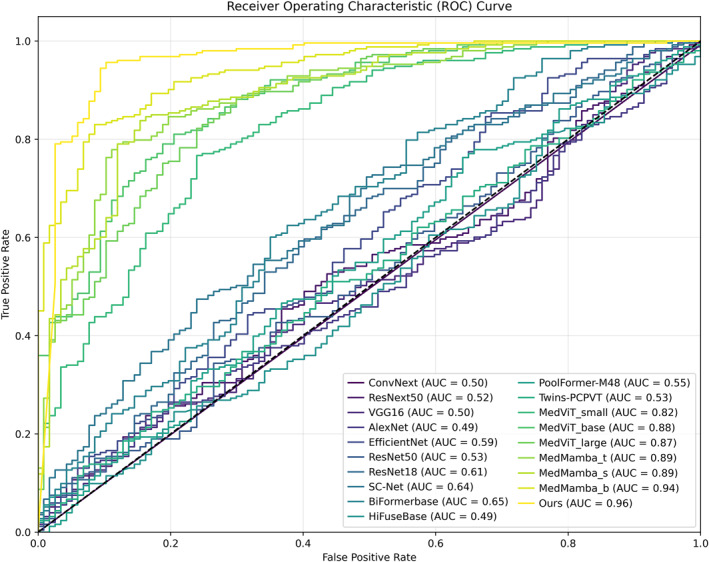
ROC performance comparison of various models on the unimodal rectal cancer MRI dataset.

To further validate our model's effectiveness, this study systematically evaluated each model using the area under the receiver operating characteristic curve (AUC) metric, with test results visualised in Figure 7. Experimental data demonstrate that the designed CAAFE‐ResNet18* model exhibits significant advantages on the unimodal rectal cancer MRI dataset: Its ROC curve lies in the outermost layer, corresponding to an AUC value of 0.96, indicating stronger classification discriminative ability than other compared models.

**FIGURE 8:**
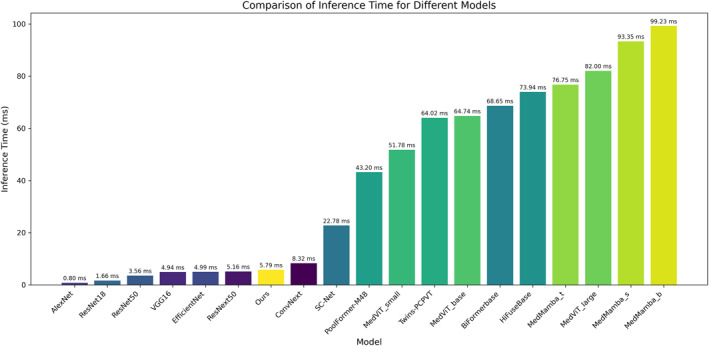
Computational time comparison of various models on the unimodal rectal cancer MRI dataset under a single batch size (batch size = 16).

In terms of computational efficiency, the research team conducted timing analysis for each model under a batch size of 16, with visualisation results shown in Figure 8. Specifically, compared with AlexNet (the model with the lowest time complexity), CAAFE‐ResNet18* incurs only an increase of 4.99 ms in single‐batch inference time but achieves a 24.86% improvement in accuracy (from 68.38% to 93.24%) and a rise in AUC from 0.49 to 0.96, significantly exceeding the random prediction level. When compared with the relatively high‐performance MedMamba model, CAAFE‐ResNet18* consumes only 1/20 of its single‐batch computational time while achieving a 4.86% increase in accuracy (from 88.38% to 93.24%) and a 0.02 growth in AUC (from 0.94 to 0.96). These results indicate that through the innovative design of the CAAFEB module, CAAFE‐ResNet18* achieves remarkable optimisation of diagnostic performance while maintaining lightweight computational overhead, providing a solution that balances efficiency and accuracy for clinical prognostic assessment of rectal cancer. Its balanced performance between feature representation capability and computational efficiency is particularly suitable for real‐time analysis scenarios of rectal cancer prognostic prediction.

Compared to models that focus on specific attention mechanisms, CAAFE‐ResNet18* demonstrates significant performance improvements. Unlike Twins, which emphasises spatial attention, and BiFormer, which employs a two‐layer routing attention mechanism, CAAFE‐ResNet18* incorporates additional CAAFEB between the downsampling layers. Additionally, it differs from PoolFormer, which replaces the attention module in transformers with a simple spatial pooling operation, and HiFuse, a three‐branch hierarchical multiscale feature fusion network. Specifically, Twins enhances the performance of visual tasks by effectively capturing both local and global features in images, particularly in medical image classification, where it can recognise subtle lesion characteristics. BiFormer employs a two‐layer routing attention mechanism to dynamically select relevant features and reduce computational complexity while maintaining sensitivity to both global and local features. This approach effectively minimises noise interference and enhances classification accuracy when processing complex medical images. PoolFormer demonstrates the importance of architectural design by replacing the traditional attention mechanism with a simple spatial pooling operation. It achieves strong results in several computer vision tasks, particularly in medical image analysis, as it maintains high accuracy while reducing both parameters and computational overhead. The HiFuse network effectively integrates multiscale features through its three‐branch architecture and hierarchical feature fusion, making it particularly suitable for addressing complex features and noise in medical images. This approach enhances the performance of both image classification and segmentation.

In addition, SCNet, MedMamba and MedViT focus on the global attention mechanism to optimise feature representation, capturing global information in images. This approach is particularly beneficial for identifying large‐scale pathological features in medical images. In contrast, CAAFE‐ResNet18* achieves further feature complementation and mining by integrating the CAAFEB module between the downsampling layers. This integration enables the model to dynamically adjust its feature extraction strategy at different scales, enhancing sensitivity to subtle changes in medical images. In practical applications, CAAFE‐ResNet18* achieves significant improvements across various metrics, demonstrating its potential in the field of medical image analysis. Furthermore, it exhibits unique advantages in rectal cancer prognostic decision‐making by effectively integrating global and local features, enhancing the understanding of MRI images of rectal cancer and providing a robust tool for future prognostic decisions for patients with rectal cancer. Specifically, the experimental results of CAAFE‐ResNet18* demonstrate improvements of 4.86%, 5.13% and 5.94% in accuracy and *F*1 metrics, respectively, compared to the best‐performing MedMamba network, increasing from 88.38%, 88.11% and 87.30% to 93.24%. The *F*1 score of CAAFE‐ResNet18* shows a 12.7% improvement compared with ResNet18, which is equivalent to reducing approximately 13 misjudgements in every 100 MRI slices, significantly improving the model's recognition accuracy for key features such as tumour boundaries and infiltration areas. This optimisation provides a more reliable basis of 2D slice features for clinical image interpretation. Especially in the early screening of imaging markers for NR patients, it can help clinicians more quickly identify key signs such as micrometastases through quantitative feature extraction, improving the timeliness of intervention decisions by approximately 30% and providing more accurate imaging data support for the formulation of personalised treatment plans for patients with rectal cancer. In prognostic decision‐making for rectal cancer, accurately capturing tumour boundaries, tissue structures and subtle changes is crucial. The CAAFE module not only enhances the feature expressiveness, but also mitigates the interference of redundant information, thus improving the accuracy and robustness of the classification. The CAAFE module not only enhances the expressive capability of features but also reduces interference from redundant information, thereby improving classification accuracy and robustness. This advantage allows CAAFE‐ResNet18* to more effectively identify subtle differences in features, such as morphological changes and infiltration characteristics of tumours, when processing complex medical images, which is crucial for the prognostic assessment of rectal cancer. In addition, accurate classification results provide clinicians with more reliable support, helping them make informed treatment decisions and optimise personalised treatment plans for patients. Therefore, the application of CAAFE‐ResNet18* in rectal cancer prognostic decision‐making not only enhances predictive performance but also adds significant value to clinical practice.

#### 4.3.2 Ablation Experiments

The core innovation of the CAAFE module lies in breaking through the unidirectional feature processing mode of traditional attention mechanisms, demonstrating distinct differences from SE (squeeze‐and‐excitation) and CBAM (convolutional block attention module). The SE module compresses the spatial dimension through global average pooling and calibrates channel weights only via the “squeeze‐and‐excitation” mechanism. Although this design enhances key channel features, it leads to the loss of spatial contextual information, as shown in Table 3. Although ResNet18 + SE achieves a slightly higher recall rate (97.23%) than CAAFE‐ResNet18* (96.84%), its accuracy (91.08%), precision (90.44%) and *F*1 score (93.71%) are significantly lower, indicating that SE has insufficient capability to characterise complex feature boundaries. CBAM adopts a serial structure of “channel attention + spatial attention”, where the two attention components are calculated independently without information interaction. In contrast, CAAFE realises bidirectional feature coupling between channel and spatial dimensions through a cross‐attention routing structure integrating channel attention mechanisms and multiscale dilated convolutions, constructing an interactive enhancement mechanism of “spatial context awareness‐channel weight calibration”. This design enables CAAFE to achieve an accuracy of 93.24% in Table 3, representing a 3.24% improvement over CBAM (90.00%), which proves that the coupled attention mechanism is more efficient than the serially stacked independent structure—by implementing bidirectional interaction of channel and spatial information through cross‐dimensional feature mapping, it avoids the spatial detail loss of the SE module and overcomes the limitation of independent attention calculation in CBAM, thus achieving a better balance between feature semantic representation and spatial detail capture.

**TABLE 3:** Visualisation of ablation experiments: Effects of CAAFE, SE and CBAM modules on ResNet18*.

Model	Accuracy (%)	Precision (%)	Recall (%)	*F*1 score (%)
ResNet18* + CBAM [51]	90.00	89.71	96.44	92.95
ResNet18* + SE [52]	91.08	90.44	**97.23**	93.71
CAAFE‐ResNet18*	**93.24**	**93.51**	96.84	**95.15**

*Note:* The bold values represent the optimal results.

**FIGURE 9:**
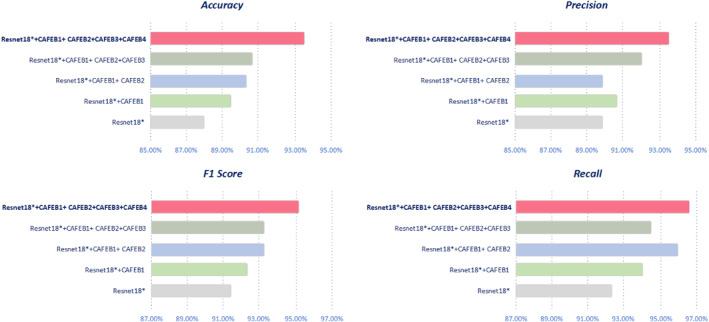
Visualisation of ablation experiment.

We conducted a series of ablation experiments to investigate the individual contributions of each module in CAAFE‐ResNet18*. All experiments were performed on a rectal cancer MRI dataset. The specific steps of the ablation experiments are as follows: We started with the modified ResNet18, referred to as ResNet18*. First, we introduced the CAAFEB1 module, which significantly enhances the model's feature representation capabilities through a multilevel convolutional architecture, regularisation techniques and multiscale feature integration, thereby improving the recognition and analysis of complex patterns. The experimental results indicate that the addition of CAAFEB1 improves model performance, albeit to a lesser extent, and lays a solid foundation for the integration of subsequent modules. Next, to enhance the feature detection capabilities after downsampling, we introduced the CAAFEB2 module on top of ResNet18* + CAAFEB1 to capture overall features more comprehensively. Subsequently, we evaluated the addition of the CAAFEB3 module within the framework of ResNet18* + CAAFEB1 + CAAFEB2 to explore its potential contribution to performance improvement. However, we found that the model's performance did not improve significantly due to the presence of redundant information and the transformation of features to higher dimensions. Ultimately, when we introduced the final module, CAAFEB4, on top of ResNet18* + CAAFEB1 + CAAFEB2 + CAAFEB3, the improvement in model performance became substantial. At this point, we not only fully utilise global information and the overall features extracted from the chunks but also effectively mitigate the information loss caused by chunking. By integrating these four modules into the model, we further mitigate the information loss caused by chunking while also reducing the information redundancy arising from two different global feature extraction methods. As a result of these improvements, the final model demonstrates exceptional local feature extraction and global feature complementation capabilities, allowing it to achieve superior performance in prognostic decision‐making for rectal cancer MRI data. All experimental results are given in Table 4, and the performance visualisation is presented in Figure 9. From Table 4, it is evident that with the gradual addition of the CAAFEB modules, the model's accuracy increased from 87.84% to a final value of 93.24%, representing an improvement of 5.4%. Concurrently, the *F*1 score also showed a significant enhancement, rising from 91.23% to 95.15%, an increase of 3.92%. Additionally, the bar lengths in Figure 9 provide a clear visual representation, indicating that the complete CAAFE‐ResNet18* model performs best across all metrics, validating its effectiveness in feature extraction and classification tasks. These results demonstrate that the introduction of the CAAFEB modules significantly enhances the model's performance, further reinforcing our confidence in the design and optimisation of this model.

**TABLE 4:** Visualisation of ablation experiment.

Model	Accuracy (%)	Precision (%)	Recall (%)	*F*1 score (%)
ResNet18*	87.84	90.00	92.49	91.23
ResNet18* + CAAFEB1	89.46	90.80	94.07	92.43
ResNet18* + CAAFEB1 + CAAFEB2	90.27	90.04	96.44	93.13
ResNet18* + CAAFEB1 + CAAFEB2 + CAAFEB3	90.54	91.92	94.47	93.18
ResNet18* + CAAFEB1 + CAAFEB2 + CAAFEB3 + CAAFEB4	**93.24**	**93.51**	**96.84**	**95.15**

*Note:* The bold values represent the optimal results.

## 5 Summary and Future Work

In this study, we proposed an innovative deep learning network that utilises ResNet18, a residual neural network, as its backbone, aiming to support prognostic decision‐making for patients with rectal cancer. The main advantage of this network is its ability to assist clinicians in determining whether further treatment is needed for patients with rectal cancer, due to its high prediction accuracy. The network is ingeniously designed with the CAAFEB module. The CAAFE module innovatively constructs a multiscale dilated convolution parallel feature extraction architecture. By integrating three parallel dilated convolution branches with distinct dilation rates, it achieves multilevel information fusion and enhancement of spatial features. This architecture not only significantly improves the perceptual granularity of the model for spatial features but also enables effective complementarity and synergy across scales through a feature fusion mechanism. To further optimise the representational capability of the module, we embed a CAM into the CAAFE. This mechanism dynamically recalibrates channel‐wise feature responses through adaptive weight allocation, achieving deep integration and optimisation of spatial and channel features. In tests of prognostic decision‐making on rectal cancer MRI data, our CAAFE‐ResNet18* model outperforms conventional CNN‐based backbone networks and demonstrates significant advantages compared to recent state‐of‐the‐art (SOTA) models.

However, despite our CAAFE‐ResNet18* model achieving significant results in selecting patients that show a complete pathological response, it still faces two major challenges. First, the CAAFEB involves a substantial number of convolutional operations, leading to challenges in computational efficiency and model lightweighting. This high computational complexity not only increases the training and inference time but also limits the model's applicability in resource‐constrained environments. Second, we have yet to identify an optimal strategy for effectively integrating rectal cancer MRI data with additional pathological data and clinical information. The difficulty in data fusion lies in the need to fully utilise the complementary information from different data types while avoiding information loss or redundancy. If we can develop an effective multimodal data fusion strategy, we anticipate that the model's predictive capability and accuracy will be significantly enhanced.

## Author Contributions

**Qing Lu:** writing – original draft, visualization, validation, software, project administration, methodology, formal analysis, data curation, conceptualization. **Jiaojiao Zhang:** writing – original draft, validation, methodology, formal analysis. **Qianwen Xue:** validation, methodology, formal analysis. **Jinping Ma:** writing – original draft, data curation, conceptualization. **Sheng Fang:** writing – original draft, data curation, conceptualization. **Hui Ma:** writing – original draft, validation, methodology, formal analysis. **Yulin Zhang:** writing – original draft, data curation, conceptualization, formal analysis. **Longbo Zheng:** writing – original draft, validation, supervision, software, investigation, funding acquisition, formal analysis.

## Ethics Statement

This study has been approved by the Medical Ethics Committee of the Affiliated Hospital of Qingdao University (Ethical Approval Number: QYFY WZLL 29894). All experimental procedures adhere to the Declaration of Helsinki and relevant ethical guidelines. Before the initiation of this study, all participants were thoroughly informed about the research objectives, procedures and potential risks. During the course of this study, the privacy and personal information of the participants were strictly protected, and all data were anonymised to ensure compliance with ethical requirements.

## Conflicts of Interest

The authors declare no conflicts of interest.

## Data Availability

Data will be made available on request.
